# Impact of the 2019 Novel Coronavirus Disease Pandemic on the Performance of a Cardiovascular Department in a Non-epidemic Center in Beijing, China

**DOI:** 10.3389/fcvm.2021.630816

**Published:** 2021-02-18

**Authors:** Jing Nan, Tong Zhang, Yali Tian, Ke Song, Qun Li, Qiang Fu, Yan Ma, Zening Jin

**Affiliations:** ^1^Department of Cardiology and Macrovascular Disease, Beijing Tiantan Hospital, Capital Medical University, Beijing, China; ^2^Tiantan Neuroimaging Center of Excellence, Beijing Tiantan Hospital, Capital Medical University, Beijing, China

**Keywords:** 2019 novel coronavirus disease, ST-segment elevation myocardial infarction, percutaneous coronary intervention, coronary angiography, Door-2-Door

## Abstract

**Background:** Knowledge of the impact of the 2019 novel coronavirus disease (COVID-19) pandemic on the performance of a cardiovascular department in a medical referral hub center from a non-epidemic area of China is limited.

**Method:** The data on the total number of non-emergency medical cares (including the number of out-patient clinic attendances, the number of patients who were hospitalized in non-intensive care wards, and patients who underwent elective cardiac intervention procedures) and emergency medical cares [including the number of emergency department (ED attendances) and chest pain center (CPC attendances), as well as the number of patients who were hospitalized in coronary care unit (CCU) and the number of patients who underwent emergency cardiac intervention procedures] before and during the pandemic (time before the pandemic: 20th January 2019 to 31st March 2019 and time during the pandemic: 20th January 2020 to 31st March 2020) in the Department of Cardiology and Macrovascular Disease, Beijing Tiantan Hospital, Capital Medical University were collected and compared.

**Results:** Both the non-emergency medical and emergency medical cares were affected by the pandemic. The total number of out-patient clinic attendance decreased by 44.8% and the total number of patients who were hospitalized in non-intensive care wards decreased by 56.4%. Pearson correlation analysis showed that the number of out-patient clinic attendance per day was not associated with the number of new confirmed COVID-19 cases and the cumulative number of confirmed COVID-19 patients in Beijing (*r* = −0.080, *p* = 0.506 and *r* = −0.071, *p* = 0.552, respectively). The total number of patients who underwent non-emergency cardiac intervention procedures decreased during the pandemic, although there were no statistically significant differences except for patent foramen ovale (PFO) occlusion (1.7 ± 2.9 vs. 8.3 ± 2.3, *p* = 0.035). As for the emergency medical cares, the ED attendances decreased by 22.4%, the total number of CPC attendances increased by 10.3%, and the number of patients who were hospitalized in CCU increased by 8.9%: these differences were not statistically significant. During the pandemic, the proportion of hospitalized patients with ST segment elevation myocardial infarction (STEMI) and non-ST segment elevation myocardial infarction (NSTEMI) significantly increased (19.0 vs. 8.7%, *p* < 0.001; 28.8 vs. 18.0%, *p* < 0.001, respectively); also, the number of primary percutaneous coronary intervention (PCI) increased by 10.3%. There was no significant difference between patients before and during the pandemic regarding the age, gender, baseline and discharge medication therapy, as well as length of stay and in-hospital mortality.

**Conclusions:** Our preliminary results demonstrate that both the non-emergency and emergency medical cares were affected by the COVID-19 pandemic even in a referral medical center with low cross-infection risk. The number of the out-patient clinic attendances not associated with the number of confirmed COVID-19 cases could be due to different factors, such as the local government contamination measures. The proportion of hospitalized patients with acute myocardial infarction increased in our center during the pandemic since other hospitals stopped performing primary angioplasty. A hub-and-spoke model could be effective in limiting the collateral damage for patients affected by cardiovascular diseases when the medical system is stressed by disasters, such as COVID-19 pandemic.

## Introduction

The 2019 novel coronavirus disease (COVID-19), caused by the novel severe acute respiratory syndrome coronavirus 2 (SARS-CoV2), has become a global pandemic since March 2020 (1), compromising the normal performance of health-care facilities in different aspects (2–5). Patients with acute or chronic cardiovascular diseases (CVD) encountered difficulties during this historical pandemic (6–9). Actually, a significant reduction of patient admissions due to different types of CVDs was observed (10–13).

Compared with other regions like Hubei province with high risk of cross-infection, the overall risk of cross-infection in Beijing was relatively low (the total number people who lives in Beijing is more than 20 million and the total confirmed number of COVID-19 infection was 580 by 31st March 2020). The reason for the lack of involvement by the pandemic in Beijing is largely attributed to the strict counter-contamination measures that local government applied during the pandemic. Those measures included wearing masks; closing public places such as restaurants, theaters, and clubs; encouraging telecommuting; etc. Most of the hospitals in Beijing stopped performing primary percutaneous coronary intervention (PCI) considering the possible risk of cross-infection. Our hospital, however, continued to perform primary PCI because we were relatively far from the center of Beijing.

Beijing Tiantan Hospital, Capital Medical University is a local medical referral center in the southwest of Beijing, with 1,650 beds; there are more than 400 out-patient clinic attendances in the Department of Cardiology and Macrovascular Disease per day, with 99 beds (including coronary care unit, CCU). We applied a detailed cardiovascular disease management system, which has been reported elsewhere for non-emergency and emergency medical cares (14, 15). In particular, patients without signs of infection and without medical contact history could receive medical service as usual after checking their body temperature. For patients with infection symptoms or with medical contact history, an isolated clinic room was prepared for them. Emergency cardiac intervention procedures like primary PCI were performed after the patients performed blood routine tests, chest computed tomography, and nucleic acid tests. For hemodynamically unstable critical patients, isolated patient wards were prepared for them before they finished the screening tests and fibrinolysis therapy was recommended for ST segment elevation myocardial infarction (STEMI) patients. None of the critical patients were hospitalized in our center during the pandemic. Hospitalized patients were also required to finish these tests before hospitalization.

Previous studies have evaluated the impact of the COVID-19 pandemic on the management of patients with CVD and acute myocardial infarction (AMI) (16–19). However, the impact of the COVID-19 pandemic on the standard performance of cardiovascular departments in hub-and-spoke organization systems, moreover in non-epidemic centers, has not been systemically evaluated so far. In this study, we investigated the impact of the COVID-19 pandemic on the normal performance of a cardiovascular department in a large non-epidemic hub center from Beijing, China.

## Materials and Methods

### Study Design and Population

In this single-center, retrospective, observational study, data on out-patient clinic attendances, emergency department (ED) attendances, chest pain center (CPC) attendances, patients who were hospitalized in our department, and patients who underwent non-emergency and emergency cardiac intervention procedures in the Department of Cardiology and Macrovascular Disease, Beijing Tiantan Hospital, Capital Medical University, which was a referral hub center for primary PCI, before and during the pandemic were collected and analyzed. The period before the pandemic was defined from 20th January 2019 to 31st March 2019 and the period during the pandemic was defined from 20th January 2020 to 31st March 2020. For the out-patient clinic, ER, and CPC attendances, patients <years of age were not included in this study. Demographic variables and clinical information, including comorbidities, length of stay (LOS), and in-hospital mortality for hospitalized patients, were retrieved from electronic medical records. The cumulative number of confirmed COVID-19 cases in Beijing was retrieved from the official website of the National Health Commission of the People's Republic of China (http://www.nhc.gov.cn). The Beijing Tiantan Hospital, Capital Medical University Review Board approved the study and waived patient consent due to the retrospective nature of the study.

### Primary and Secondary Outcomes

The primary outcomes evaluated were non-emergency medical cares including the attendances to the out-patient clinic, the number of patients who were hospitalized in the non-intensive care wards, and the number of patients who underwent elective cardiac intervention procedures. Secondary outcomes were emergency medical cares including the attendances to the ED and CPC, the total number of patients who were hospitalized in coronary care unit (CCU), and the number of patients who underwent primary PCI.

### Statistical Analysis

Continuous variables were expressed as means ± standard deviation or median (25 and 75% quartiles) according to the normality of distribution. We performed a Student *t*-test or Mann–Whitney *U*-test as appropriate. Categorical variables were expressed as numbers (percentages) and compared using Pearson's chi-squared tests. Pearson correlation analysis was used to evaluate the associations of the out-patient clinic and ED and CPC attendances with the cumulative number of confirmed COVID-19 cases in Beijing. SPSS 22 (Chicago) was used for all statistical analyses, and a *p* < 0.05 was considered statistically significant.

## Results

### Comparison of Non-emergency Medical Cares Before and During the Pandemic

**Figure 1:**
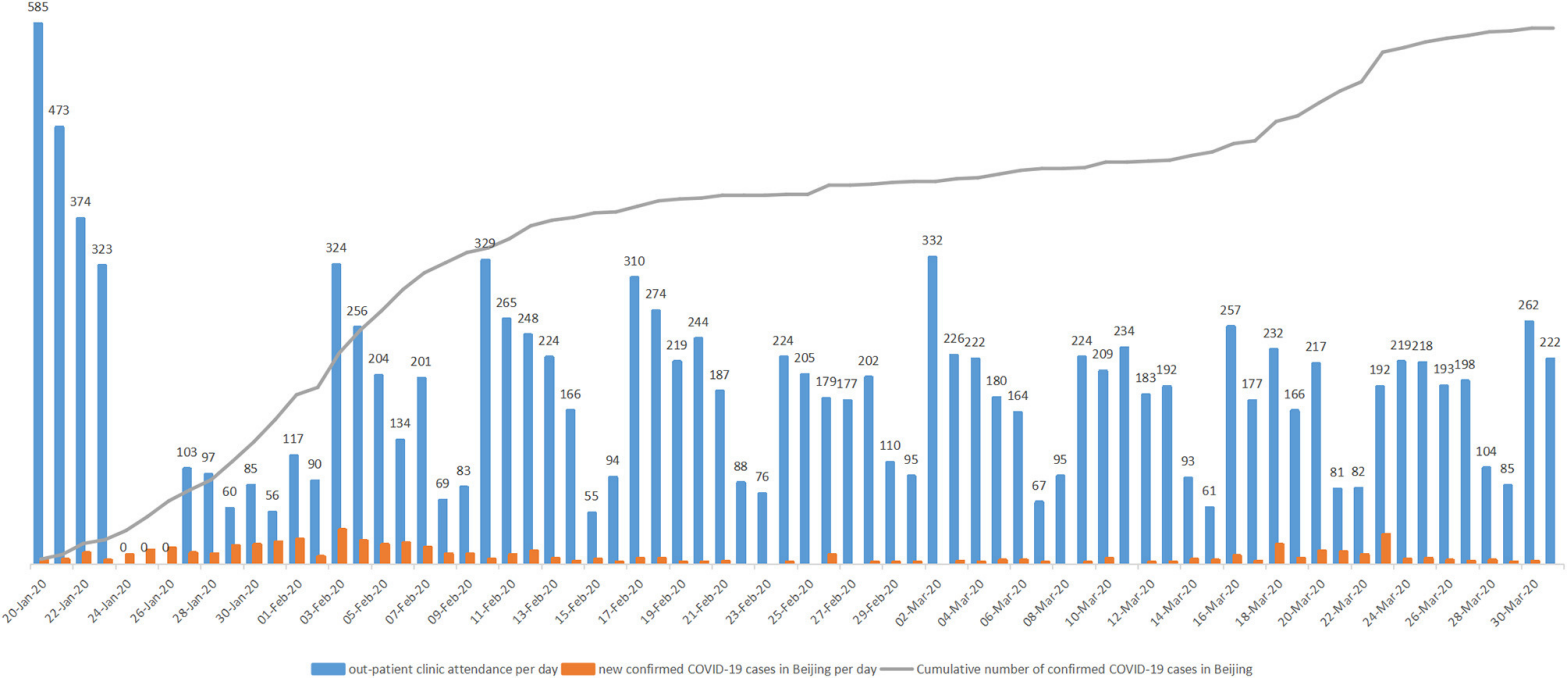
The association of the total number of out-patient attendance per day with the new confirmed COVID-19 patients and cumulative number of confirmed COVID-19 patients in Beijing.

The number of out-patient clinic attendances per month during the pandemic is decreased compared with the number of out-patient clinic attendances per month before the pandemic (4330.7 ± 1884.4 vs. 7847.0 ± 3686.3, *p* = 0.215) although this is not significantly different. Pearson correlation analysis showed that the number of out-patient clinic attendances per day was not associated with the number of new confirmed COVID-19 patients in Beijing and the cumulative number of confirmed COVID-19 patients in Beijing (*r* = −0.080, *p* = 0.506 and *r* = −0.071, *p* = 0.552, respectively) (Figure 1). There was no significant difference in the number of patients who underwent elective cardiac intervention procedures per month during the pandemic when comparing the number of patients before the pandemic in coronary angiography (74.0 ± 32.9 vs. 118.3 ± 62.1, *p* = 0.336), PCI (51.3 ± 28.4 vs. 74.0 ± 40.0, *p* = 0.468), permanent pacemaker implantation (2.3 ± 2.1 vs. 5.0 ± 4.4, *p* = 0.393), and radiofrequency ablation (0.3 ± 0.6 vs. 2.7 ± 1.5, *p* = 0.069) except for patent foramen ovale (PFO) occlusion (1.7±2.9 vs. 8.3±2.3, *p* = 0.035).

### Comparison of the Emergency Medical Cares Before and During the Pandemic

There is no significant difference between the number of ED attendances per month during the pandemic compared with the number of ED attendances per month before the pandemic (8288.7 ± 3294.9 vs. 11636.0 ± 350.3, *p* = 0.923) as well as the number of CPC visits per month (569.7 ± 334.3 vs. 543.3 ± 294.6, *p* = 0.295). The number of patients who underwent primary PCI per month is increased during the pandemic compared with the number of patients before the pandemic (20.3 ± 5.5 vs. 18.0 ± 6.1, *p* = 0.648). However, these differences are not statistically significant.

### Comparisons of the Total Number and Clinical Outcomes of Patients Who Were Hospitalized Before and During the Pandemic

Comparisons of the total number and clinical outcomes of patients who were hospitalized before and during the pandemic are shown in Table 1. Compared with the number of hospitalized patients before the pandemic, the total number of patients who were hospitalized in the non-intensive care wards per month is decreased (113.7 ± 82.1 vs. 173.3 ± 43.0, *p* = 0.327), whereas the total number of patients who were hospitalized in CCU per month during the pandemic is increased (53.0 ± 4.6 vs. 48.7 ± 4.5, *p* = 0.308), but there is no significant difference. Also, there are no significant differences with respect to gender distribution, age, medication therapy on admission and discharge, reasons for admission, LOS, and in-hospital mortality between hospitalized patients before and during the pandemic. The reasons for mortality before the pandemic were as follows: cardiac shock (*n* = 4), cardiac rupture (*n* = 3), respiratory failure (*n* = 1), and coronary perforation (*n* = 1), and the reasons for mortality during the pandemic were as follows: cardiac shock (*n* = 4), cardiac rupture (*n* = 2), and respiratory failure (*n* = 1). Compared with the hospitalized patients before the pandemic, the proportion of hospitalized patients with ST segment elevation myocardial infarction (STEMI) and non-ST segment elevation myocardial infarction (NSTEMI) is significantly increased during the pandemic (19.0 vs. 8.7%, *p* < 0.001; 28.8 vs. 18.0%, *p* < 0.001, respectively). The proportions of hospitalized patients with unstable angina pectoris (38.6 vs. 48.8%, *p* = 0.005), PFO (3.2 vs. 6.9%, *p* = 0.026), and others (0.6 vs. 4.0%, *p* = 0.003) are significantly decreased during the pandemic, while the proportions of hospitalized patients with stable angina pectoris (1.9 vs. 3.2%, *p* = 0.375), heart failure (4.7 vs. 8.1%, *p* = 0.066), and arrhythmia (3.2 vs. 2.4%, *p* = 0.511) are not significantly different.

**Table 1:** Comparison of the total number, clinical characteristics, and clinical outcomes in hospitalized patients before and during the COVID-19 pandemic.

	**Before the pandemic**	**During the pandemic**	***P*-value**
**Number of hospitalized patients per month**			
Non-intensive care wards	120.0 ± 59.6	52.3 ± 34.9	0.165
Coronary care unit	36.7 ± 17.2	40.7 ± 417.1	0.789
**Baseline clinical characteristics in hospitalized patients**			
Male gender, *n* (%)	324 (64.0)	201 (63.6)	0.940
Age (years), median (25–75th percentiles)	64 (51–76)	66 (51.3–76)	0.596
Current smoker, *n* (%)	160 (31.6)	95 (30.1)	0.698
**Reasons for hospitalization**, ***n*** **(%)**			
STEMI	44 (8.7)	60 (19.0)	<0.001
NSTEMI	91 (18.0)	91 (28.8)	<0.001
UAP	247 (48.8)	122 (38.6)	0.005
SAP	16 (3.2)	6 (1.9)	0.375
PFO	35 (6.9)	10 (3.2)	0.026
Arrhythmia	41 (8.1)	15 (4.7)	0.066
Heart failure	12 (2.4)	10 (3.2)	0.511
Other reasons	20 (4.0)	2 (0.6)	0.003
**Medications in hospitalized patients on admission**, ***n*** **(%)**			
ACEI/ARB	88 (17.4)	52 (16.5)	0.775
β receptor blocker	33 (6.5)	18 (5.7)	0.659
Statins	184 (36.4)	110 (34.8)	0.708
Antiplatelet therapy	188 (37.2)	99 (31.3)	0.098
**Pharmaceutical treatment in hospitalized patients before discharge**, ***n*** **(%)**			
AECI/ARB	185 (36.6)	105 (33.2)	0.368
β receptor blocker	115 (22.7)	84 (26.6)	0.211
Statins	466 (92.1)	286 (90.5)	0.443
Antiplatelet therapy	468 (92.5)	302 (95.6)	0.104
**Clinical outcomes**			
Length of stay in hospital (days)	8 (7–10)	8 (7–10)	0.300
In-hospital mortality, *n* (%)	9 (1.8)	7 (2.2)	0.796

*STEMI, ST-segment elevation myocardial infarction; NSTEMI, non-ST-segment elevation myocardial infarction; UAP, unstable angina pectoris; SAP, stable angina pectoris; PFO, patent foramen ovale; ACEI, angiotensin-converting enzyme inhibitor; ARB, angiotensin receptor blocker. Values are n, mean ± SD, n (%), or median (25–75th percentiles)*.

### Results of Relative Changes in the Number of Non-emergency and Emergency Medical Care in Our Center Before and During the Pandemic

The result are shown in Table 2. As compared with the number before the pandemic, the number of non-emergency medical care decreased (out-patient clinic attendances are decreased by 44.8%, the total number of patients who were hospitalized in the non-intensive care wards is decreased by 22.4%, and patients who underwent elective cardiac intervention procedures are all decreased); however, the number of patients who needed emergency medical care is increased (CPC attendances is increased by 10.3%, the number of patients who were hospitalized in CCU is increased by 8.9%, and the number of patients who underwent primary PCI is increased by 13.0%) except for the attendances to the ED, which is decreased by 22.4%.

**Table 2:** Relative change of total number of non-emergency and emergency medical care before and during the pandemic.

	**Before the pandemic**	**During the pandemic**	**Relative change**
**Non-emergency medical care**			
Total number of patients who were hospitalized in internal medicine wards	360	157	−56.4%
Total number of out-patient clinic visits	23,541	12,992	−44.8%
Total number of patients who underwent coronary angiography	355	222	−37.5%
Total number of patients who underwent percutaneous coronary intervention (PCI)	222	154	−30.6%
Total number of patients who underwent permanent pacemaker implantation	15	7	−53.3%
Total number of patients who underwent radio-frequency catheter ablation	8	1	−87.5%
Total number of patients who underwent patent foramen ovale occlusion	25	5	−80.0%
**Emergency medical care**			
Total number of emergency department visits	44,908	34,866	−22.4%
Total number of chest pain center visits	2,026	2,235	+10.3%
Total number of patients who were hospitalized in coronary care unit	146	159	+8.9%
Total number of patients who underwent primary PCI	54	61	+13.0%

## Discussion

The key findings of this retrospective study indicated that the COVID-19 pandemic caused impact on the normal performance of a cardiovascular department both in non-emergency medical cares and emergency medical cares.

The first finding of this study is that the number of patients who needed non-emergency medical cares including out-patient clinic, non-intensive care ward hospitalization, and non-emergency cardiac intervention procedures decreased during the pandemic. As a regional medical hub-and-spoke organization with relatively large volume of patients, non-emergency cares were decreased during the COVID-19 pandemic. This result is quite similar to other studies. As a matter of fact, the reduction in out-patient clinic attendances was also observed during the severe acute respiratory syndrome (SARS) and the Middle East respiratory syndrome (MERS) epidemics (20–23). In this study, we also explored the association between the number of out-patient clinic attendances and the number of infection patients by Pearson analysis; however, no significant correlation was found between them. This result reveals that the reduction of non-emergency medical cares in low-risk regions like Beijing might not be associated with the number of confirmed COVID-19 cases but with other factors like local government quarantine protocol. The reduction of patients who underwent non-emergency cardiac intervention procedures during the COVID-19 pandemic has also been observed in multiple studies. Elliott et al. demonstrated a 56% reduction in elective coronary angiography (CA) and PCI (24). Furthermore, the number of patients who underwent electrophysiology procedures and those with structural heart disease who underwent invasive procedures decreased (25–27). These results are similar to our study. However, the number of PFO occlusion in our center significantly decreased while there was no statistically significant differences in other procedures when comparing with the procedure number before the pandemic. There are many reasons for this. First of all, most of the patients who underwent PFO occlusion in our center came from other provinces. The contamination protocol is stricter for them than local patients. Secondly, the number of other procedures including pacemaker implantation and RFCA were limited, which may affect the statistics and might explain this situation. The underlying reasons for the reduction of non-emergency medical cares are complicated during the pandemic: fear of cross-infection, greater use of telemedicine, lack of medical resources for non-communicable disease, possible lower incidence rate of cardiovascular disease due to the lower physical stress and different diet activity, and increased number of financially vulnerable patients who could not pay for medications or clinical follow-up during the socioeconomic depression could all be associated with this phenomenon (28–33).

The second finding of this study is that the number of emergency medical cares was also affected during the pandemic even in a medical referral hub. For emergency medical cares, the number of CPC attendances, the number of patients who were hospitalized in CCU, and those who underwent primary PCI increased in our center during the pandemic, except for the number of ED attendances, which was decreased. First of all, the reduction of the number of ED attendances is consistent with previous studies. The Attend Study compared the daily ED attendances before and during the pandemic and observed a reduction by 30% daily ED visits (34). Franchini et al. described the impact of the COVID-19 outbreak on an urban major-hospital ED attendance in Italy and observed a reduction in ED attendance by 37% (35). Hartnett et al. (36) observed a 42% of reduction in ED visits in the USA. Boserup et al. (37) also reported a significant reduction of ED visits during the pandemic especially in high-risk regions. The reason for the reduction of ED visits is believed to be associated with fear of cross-infection, unawareness of more critical illnesses than COVID-19, as well as lower physical stress and lower rate of accidents (38–40). In our study, the CPC attendances were increased in our center during the pandemic, which was not consistent with a recent report that demonstrated that the number of STEMI cases reported to China Chest Pain Centers was reduced during the COVID-19 pandemic (41). It is worthy to note that as a multi-center analysis, that study enrolled different hospitals from several different places in China that had different cross-infection risk and cardiovascular management protocols. Also, we observed that in our center, the number of primary PCI was increased; this result was not consistent with the results from other studies worldwide (41–49). There are several explanations for this difference. First, the cross-infection risk in Beijing was relatively lower than other regions. Second, Beijing Tiantan Hospital continued to perform primary PCI as a hub-and-spoke organization when many hospitals stopped performing primary PCI and transferred patients to our center during the pandemic in Beijing.

The third finding of this study is that the number of patients who were hospitalized for STEMI and NSTEMI increased significantly, and patients who were hospitalized for unstable angina pectoris, PFO, and others decreased significantly. The number of patients who were hospitalized for other conditions, like stable angina pectoris, heart failure, and arrhythmia, decreased, but there were no statistically significant differences. This result is not similar with previous studies. Folino et al. (42) observed a significant reduction in NSTEMI but not for STEMI patients admitted to the coronary care unit during the COVID-19 pandemic. De Filippo et al. (43) also observed a reduced admission for acute coronary syndrome (ACS) during the pandemic. Braiteh et al. (44) reported a 40.7% decrease in ACS (STEMI and NSTEMI) presentation in upstate New York. The reasons for this difference between our study and theirs are as follows: first, our study was single hub-and-spoke experience where AMI patients were transferred from other hospitals. Second, as we mentioned above, the overall cross-infection risk in Beijing was lower than those studies.

The fourth finding of our study is that both the LOS and in-hospital mortality remained unchanged for hospitalized patients during the COVID-19 pandemic when comparing before the pandemic. This result is similar to previous studies. Daoulah et al. (45) enrolled STEMI patients from 16 centers during the COVID-19 pandemic and found that there were no differences with respect to in-hospital events and the LOS for these patients. Fileti et al. (50) evaluated the impact of the COVDI-19 pandemic on in-hospital outcome for ACS patients who underwent coronary invasive procedures, showing that both the percutaneous coronary intervention procedural success rate and in-hospital mortality were not different.

There are some limitations in this study. First of all, this is a single-center retrospective study. Second, the severity of the COVID-19 situation in Beijing is not like other provinces, so the result of our study is not suitable for all facilities. Third, the treatment strategy for AMI patients (primary PCI or fibrinolysis therapy for STEMI patients) might be different in other hospitals. Fourth, the major limitation of this retrospective study is represented by the fact that no adjustment was made, using appropriate statistical methodology, for potential confounders, affecting the validity of the results.

Our study shows that a hub-and-spoke model could be effective in limiting the collateral damage for patients affected by cardiovascular diseases when the medical system is stressed by disasters, such as pandemics or wars.

## Data Availability Statement

The original contributions presented in the study are included in the article/supplementary material, further inquiries can be directed to the corresponding author.

## Author Contributions

JN, TZ, KS, QF, YM, QL, and ZJ were responsible for the conception and design of the article, the implementation and feasibility analysis of the research, and the analysis and interpretation of the results. JN, TZ, and YT were responsible for data collection. JN, TZ, YT, KS, QL, YM, and QF were responsible for statistical processing and writing of the paper. ZJ were responsible for the quality control, review, supervision, and management of the article. All authors read and approved the final manuscript.

## Conflict of Interest

The authors declare that the research was conducted in the absence of any commercial or financial relationships that could be construed as a potential conflict of interest.
